# Author Correction: Estrogen-related receptor gamma functions as a tumor suppressor in gastric cancer

**DOI:** 10.1038/s41467-018-06061-z

**Published:** 2018-08-31

**Authors:** Myoung-Hee Kang, Hyunji Choi, Masanobu Oshima, Jae-Ho Cheong, Seokho Kim, Jung Hoon Lee, Young Soo Park, Hueng-Sik Choi, Mi-Na Kweon, Chan-Gi Pack, Ju-Seog Lee, Gordon B. Mills, Seung-Jae Myung, Yun-Yong Park

**Affiliations:** 1 ASAN Institute for Life Sciences, ASAN Medical Center, University of Ulsan College of Medicine, Seoul, 05505 Republic of Korea; 2 Department of Convergence Medicine, University of Ulsan College of Medicine, Seoul, 05505 Republic of Korea; 3 Department of Systems Biology, MD Anderson Cancer Center, Houston, TX 77030 USA; 4 Department of Biological Sciences, Dong-A University, Busan, 49315 Republic of Korea; 5 Division of Genetics, Cancer Research Institute, Kanazawa University, Kanazawa, 920-8641 Japan; 6 Department of Surgery, Yonsei University College of Medicine, Seoul, 03722 Republic of Korea; 7 Aging Research Institute, Korea Research Institute of Bioscience and Biotechnology, Daejeon, 34141 Republic of Korea; 8 Department of Gastroenterology, University of Ulsan College of Medicine, Seoul, 05505 Republic of Korea; 9 Department of Pathology, University of Ulsan College of Medicine, Seoul, 05505 Republic of Korea; 10 National Creative Research Initiatives Center for Nuclear Receptor Signals and Hormone Research Center, School of Biological Sciences and Technology, Chonnam National University, Gwangju, 61186 Republic of Korea

Correction to: *Nature Communications*; 10.1038/s41467-018-04244-2; published online 15 May 2018

The original version of this Article contained errors in Figs. 4, 5, and 6. In Fig. 4d, the *x*-axis label incorrectly read ‘blank, +, blank, +’, and in Fig. 5e, the bars of the second graph from the left were coloured blue-orange-blue-orange. Both of these errors have been fixed in the PDF and HTML versions of the Article. Furthermore, in Fig. 6a, the right-hand image of AGS cells treated with 5 µM DY131 was inadvertently replaced with a duplicate of the left-hand image. The correct version of this figure panel appears below. For transparency, the error has not been corrected in the PDF or HTML versions of the Article.

**Fig. 5:**
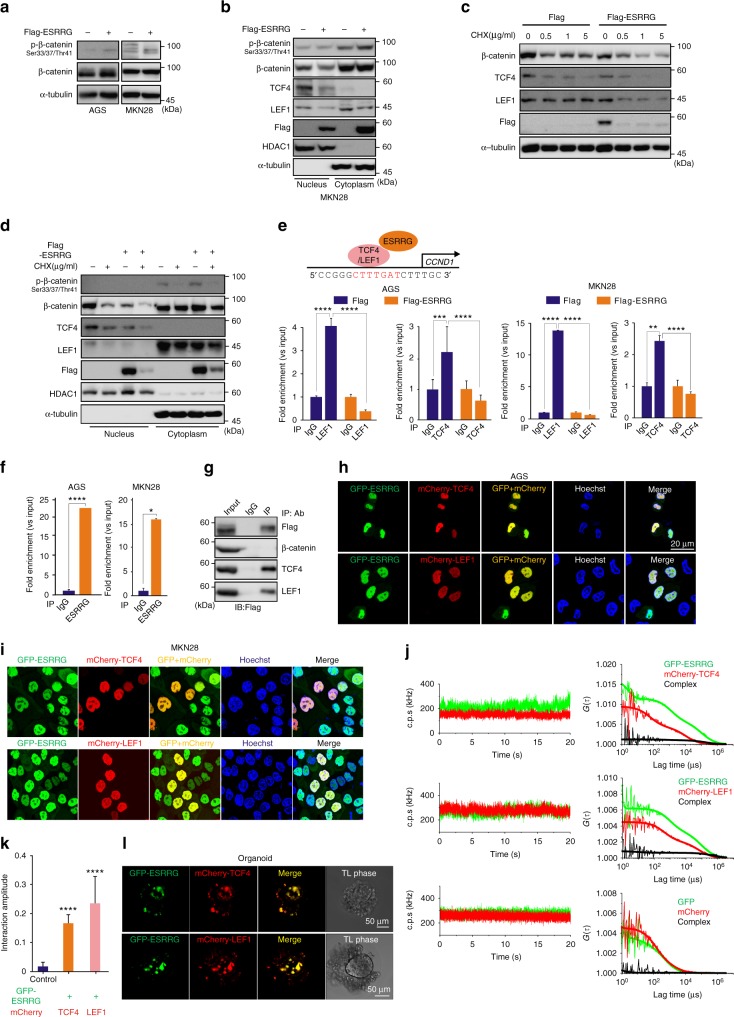
▓

**Fig. 6:**
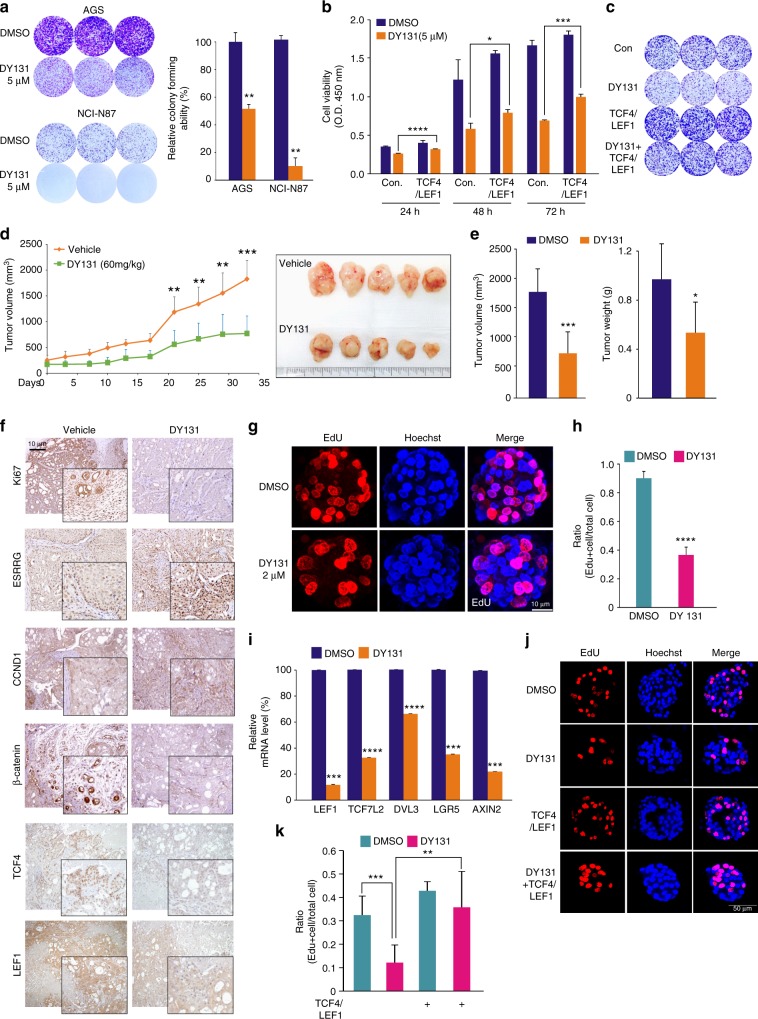
▓

