# Supplementary material for: Estrogen-related receptor gamma functions as a tumor suppressor in gastric cancer
Source: Nat Commun. 2018 May 15;9:1920. doi: 10.1038/s41467-018-04244-2 (PMC5954140; doi:10.1038/s41467-018-04244-2)

Supplementary Information for

Estrogen-related receptor gamma functions as a tumor suppressor in gastric cancer

Myoung-Hee Kang[1,2,3], Hyunji Choi[4], Masanobu Oshima[5], Jae-Ho Cheong[6], Seokho Kim[7], Jung Hoon Lee[8], Young Soo Park[9], Hueng-Sik Choi[10], Mi-Na Kweon[2], Chan-Gi Pack[1,2], Ju-Seog Lee[3], Gordon B. Mills[3], Seung-Jae Myung[1,2,8], Yun-Yong Park[1,2]

Affiliation

[1]ASAN Institute for Life Sciences, ASAN Medical Center; [2]Department of Convergence Medicine, University of Ulsan College of Medicine, Seoul, 05505, Republic of Korea; [3]Department of Systems Biology, MD Anderson Cancer Center, Houston, TX, 77030, USA,[4]Department of Biological Sciences, Dong-A University, Busan, 49315, Republic of Korea; [5]Division of Genetics, Cancer Research Institute, Kanazawa University, Kanazawa, 920-8641, Japan; [6]Department of Surgery, Yonsei University College of Medicine, Seoul, 03722, Republic of Korea; [7]Anging Research Institute, Korea Research Institute of Bioscience and Biotechnology, Daejeon, 34141, Republic of Korea; [8]Department of Gastroenterology, University of Ulsan College of Medicine, Seoul, 05505, Republic of Korea; [9]Department of Pathology, University of Ulsan College of Medicine, Seoul, 05505, Republic of Korea; [10]National Creative Research Initiatives Center for Nuclear Receptor Signals and Hormone Research Center, School of Biological Sciences and Technology, Chonnam National University, Gwangju, 61186, Republic of Korea.

Supplementary Fig. 1

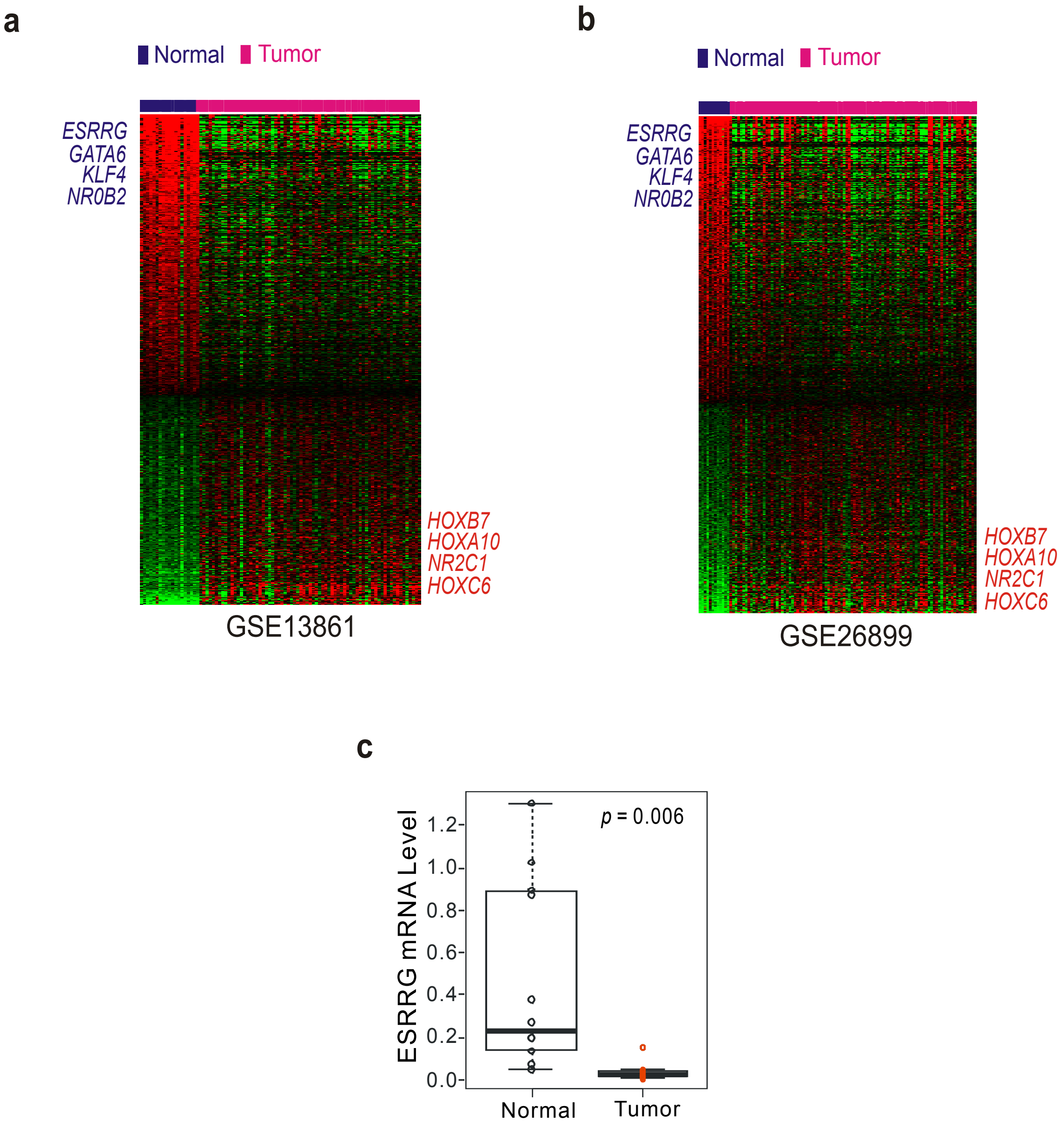

Supplementary Fig. 1 | ESRRG expression in human gastric cancer

a and b, 521 genes were visualized depending on gene expression level in indicated patients cohort. Genes involved in transcription are written in blue and red text. c, ESRRG mRNA expression in gastric cancer patients (AMC cohort). The *p* values were calculated by Student *t*-test.

Supplementary Fig. 2

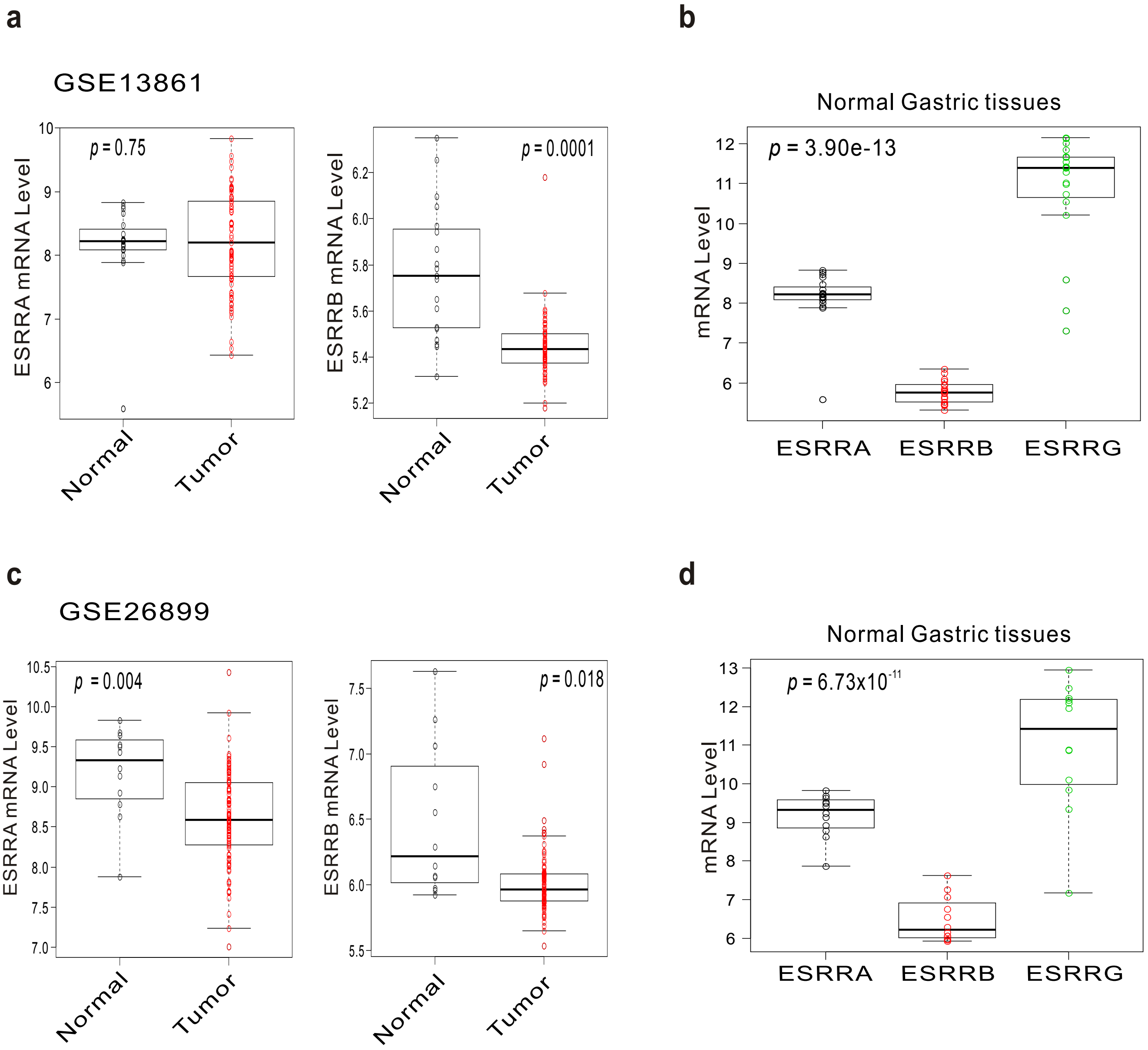

Supplementary Fig. 2 | ERRs isofom expression in human gastric cancer

a and c, ESRRA and ESRRB expression in human gastric cancer patients. The *p* values were calculated by Student t-test.b and d, Expression of three isoforms (ESRRA, ESRRB and ESRRG) of estrogen related receptors. The *p* value was calculated by ANOVA.

Supplementary Fig. 3

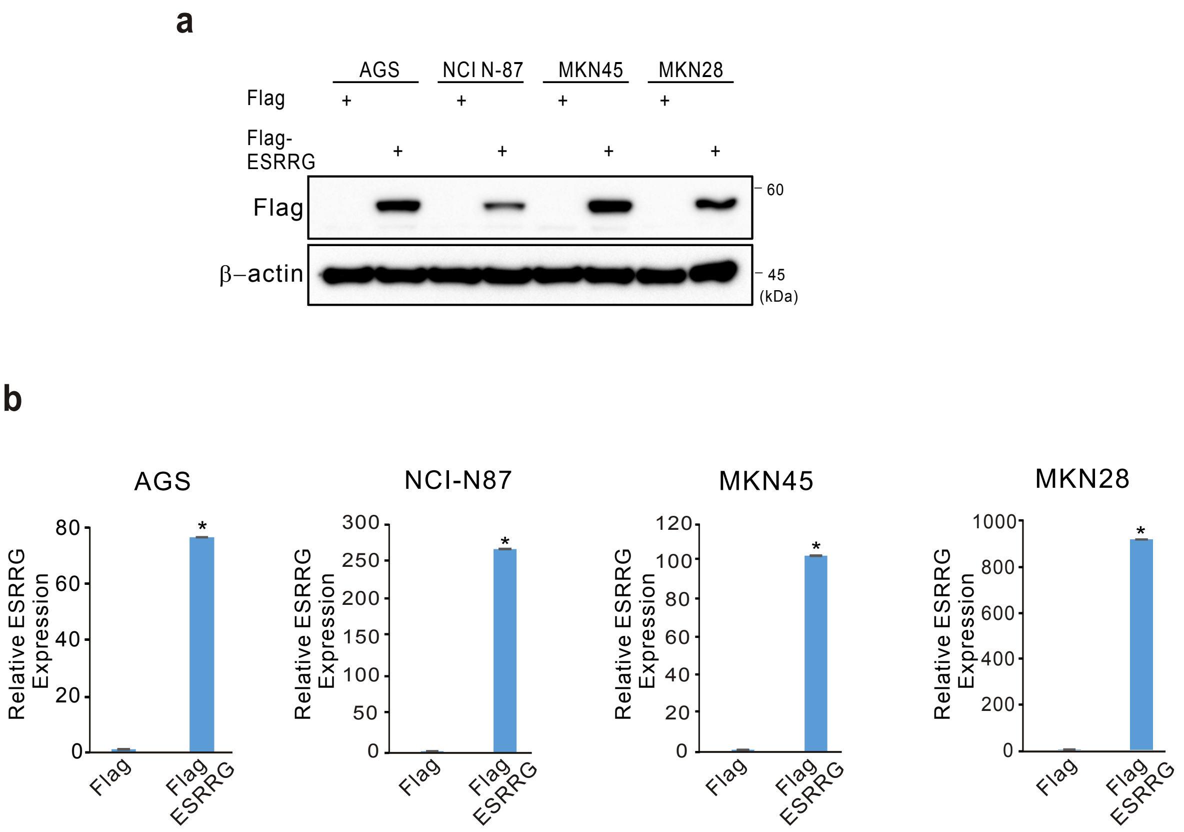

Supplementary Fig. 3 | ESRRG over-expression using lenti-virus system in gastric cancer cells

a and b, Indicated gastric can cells were stably transfected with ESRRG lenti-viral vector or control lenti-vial vector (Con). Protein lysates from infected cell lines were analyzed by western blot with indicated antibody (a).qRT-PCR using gene-specific primers as indicated (b). Student t-test (two-tailed) was applied to estimate the significance of gene expression changes: *, $p < 0.001$

Supplementary Fig. 4

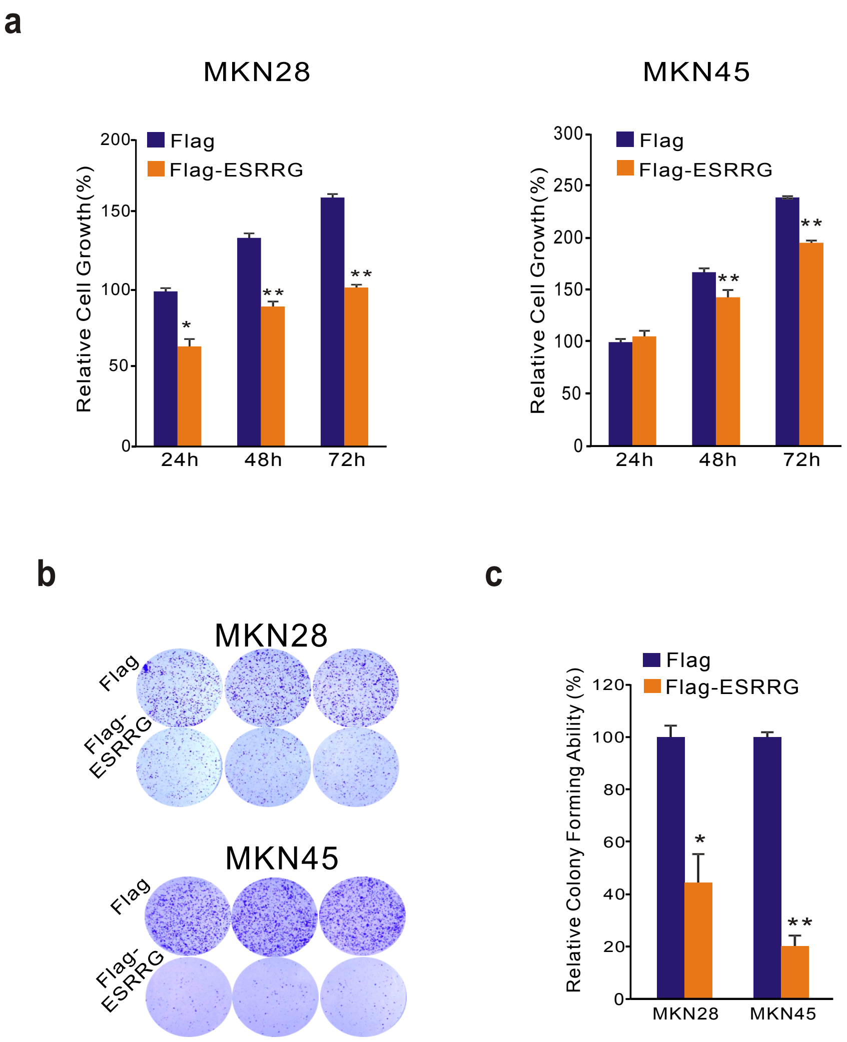

* p<0.005 **p<0.001

Supplementary Fig. 4 | ESRRG over-expression inhibits gastric cancer cell proliferation

a, Indicated gastric cancer cells were stably infected with ESRRG lenti-viral vector (Flag-ESRRG) or control lenti-vial vector (Flag). The cells were used for proliferation assay (CCK8) at the indicated time point. b and c, Infected cells were used for colony formation assay and quantified using image analyzer. Student t-test (two-tailed) was applied to estimate the significance of gene expression changes: *, $p < 0.005$, **, $p < 0.001$

Supplementary Fig. 5

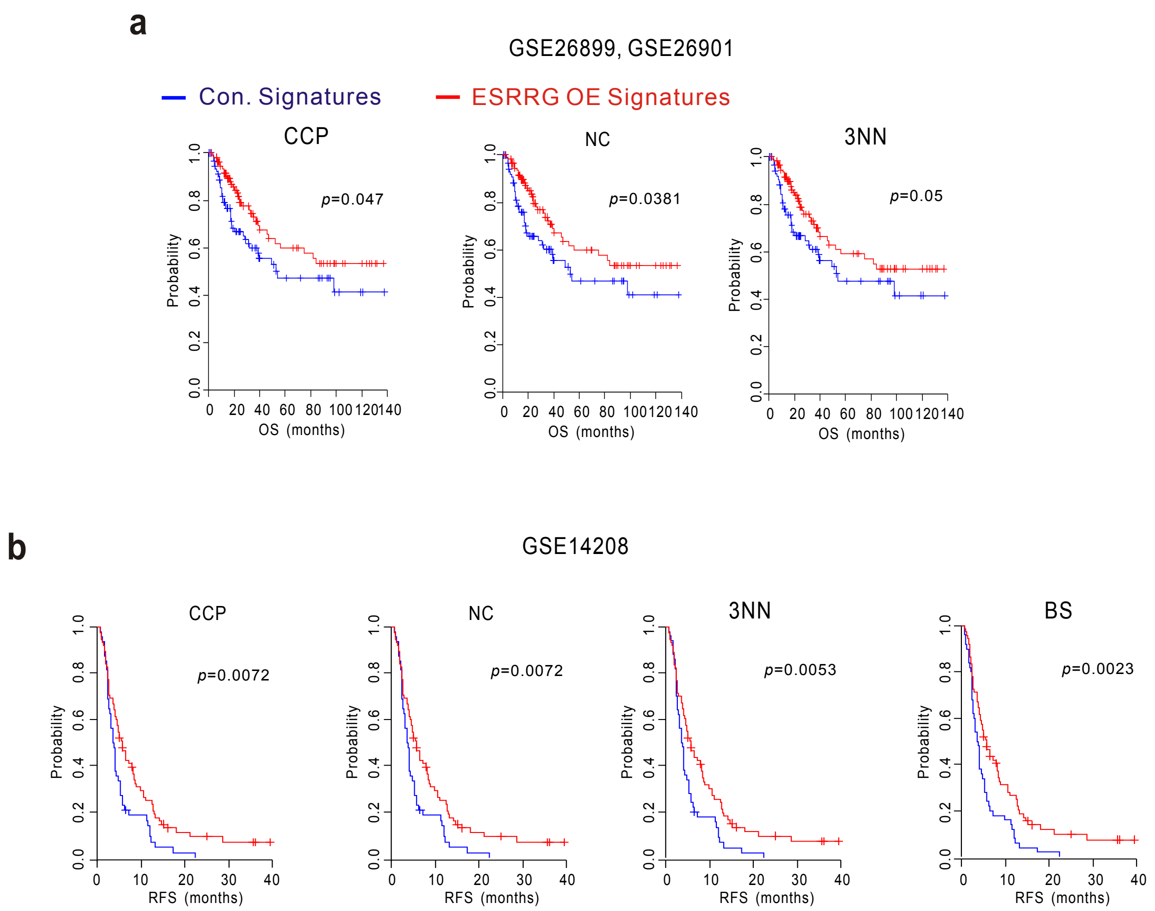

Supplementary Fig. 5| Kaplan-Meier plots of gastric cancer patients by using ESRRG gene signatures as classifier.

a-b, Kaplan-Meier plots of indicated gastric cancer patients predicted by using EERRG gene signatures from gene expression profile. The differences between groups were significant as indicated (log-rank test). Compound covariate predictor (CCP), one nearest neighbor (1NN), three nearest neighbor (3NN), nearest centroid (NC), support vector machines (SVM), linear discriminator analysis (LDA) and bayesian(BS).

Supplementary Fig. 6

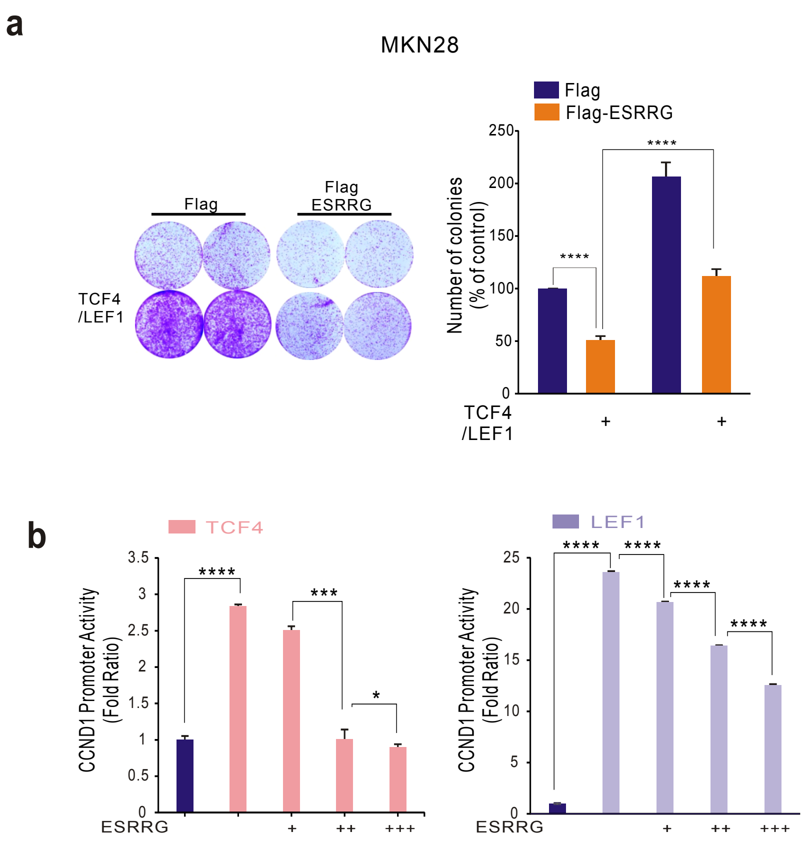

Supplementary Fig. 6 | ESRRG function on Wnt-signaling in gastric cancer cell

a, Indicated gastric cancer cells were stably infected with ESRRG lenti-viral vector (Flag-ESRRG) or control lenti-vial vector (Flag) and transfected with indicated cDNA constructs. Infected cells were used for colony formation assay and quantified using image analyzer. b, MKN28 cells were also used reporter assay with CCND1 promoter and ESRRG cDNA. Student t-test (two-tailed) was applied to estimate the significance of gene expression changes: *, $p < 0.05$; **, $p < 0.01$; ***, $p < 0.005$;****, $p < 0.001$

Supplementary Fig. 7

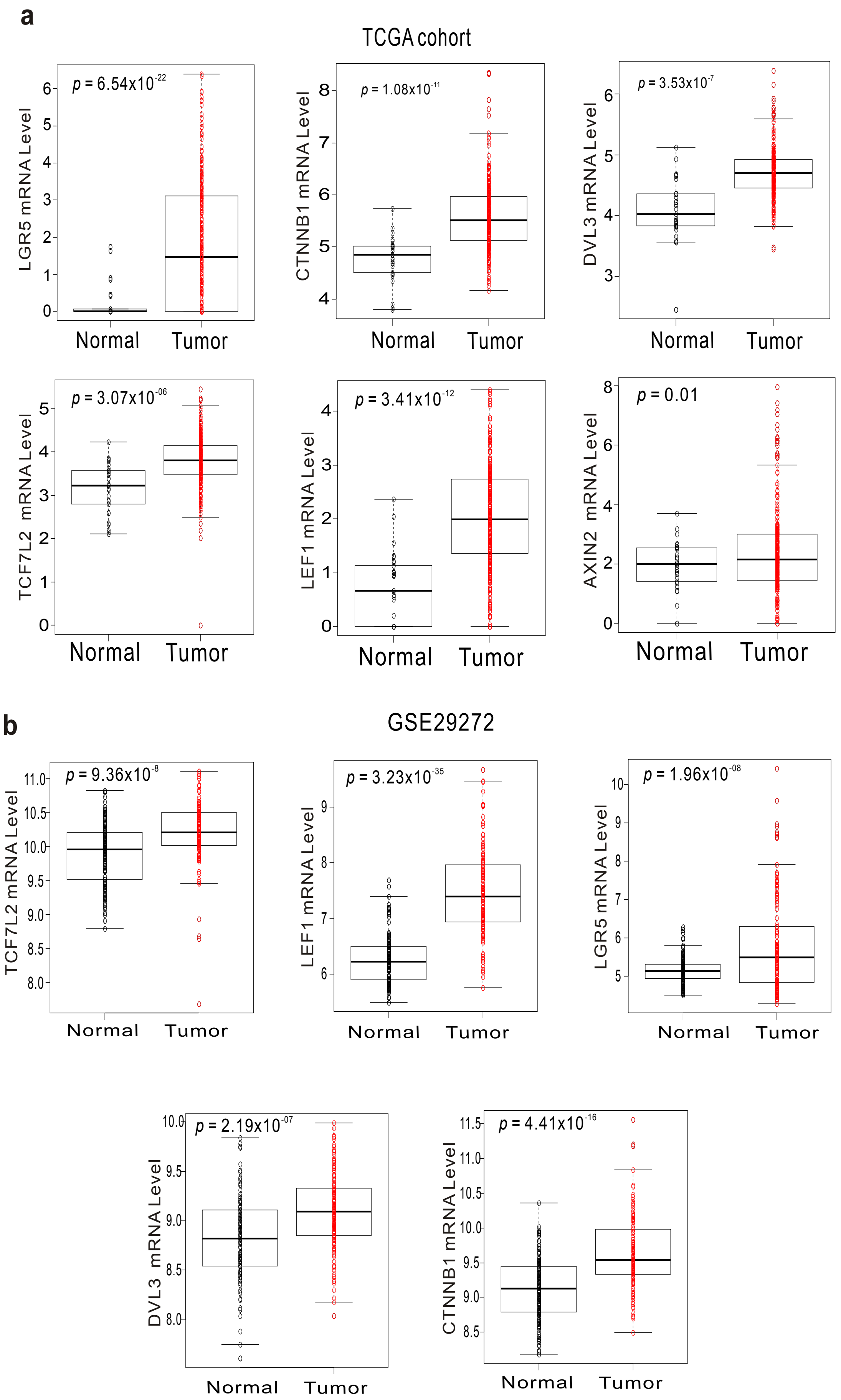

Supplementary Fig. 7 | Wnt-t target genes in human gastric cancer

a and b, Wnt-signlaing associated genes expression in human gastric cancer patients (TCGA and GSE29272). The *p* values were calculated by Student t-test.

Supplementary Fig. 8

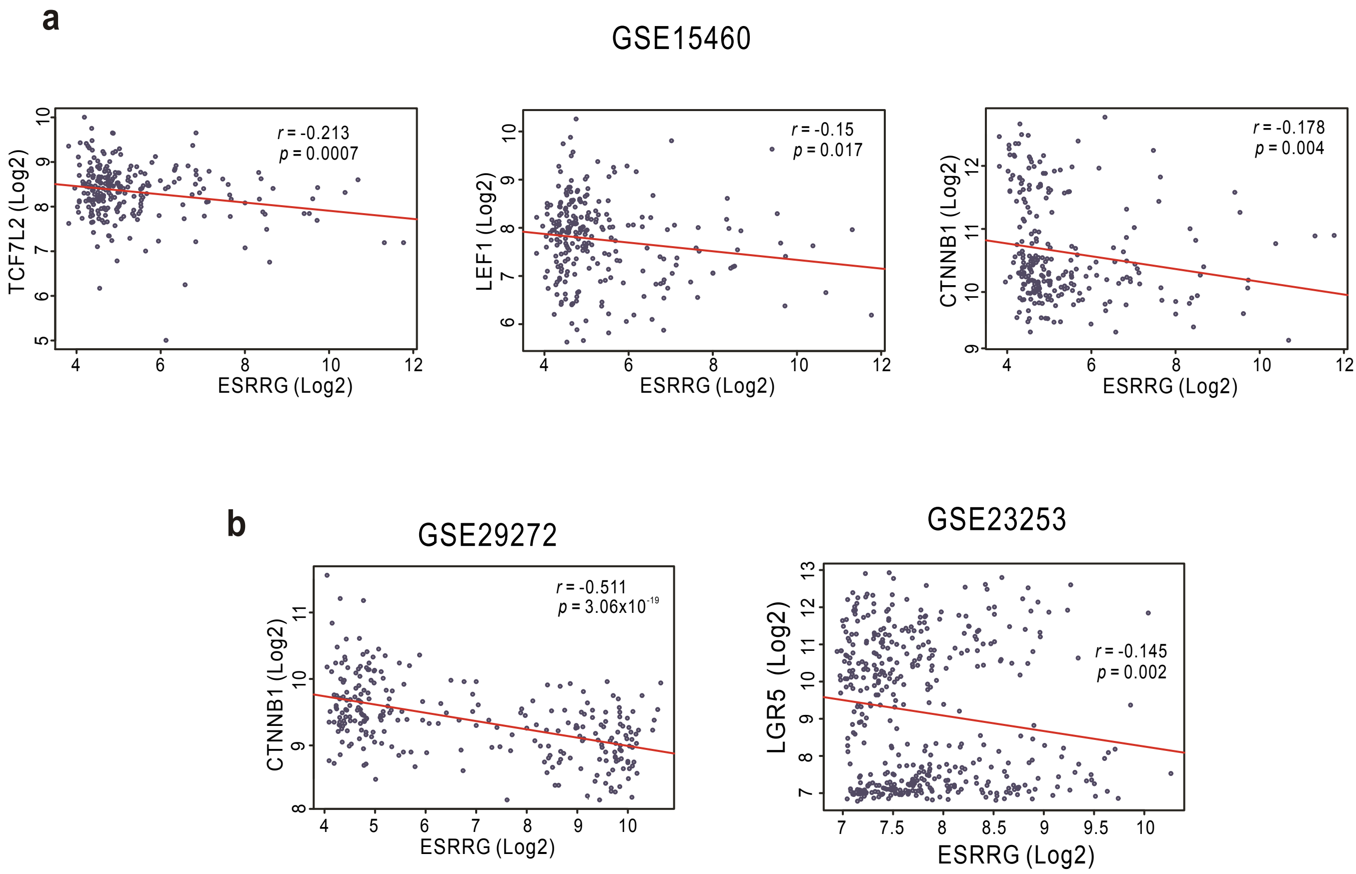

Supplementary Fig. 8 | ESRRG and Wnt-target genes correlation in gastric cancer patients

a and b, Correlation of ESRRG and Wnt-target genes expression in gastric cancer patient cohort.
Scatter plots between ESRRG and Wnt-target genes in GSE154601, GSE29272 and GSE26253 based on Pearson's correlation co-efficient value

Supplementary Fig. 9

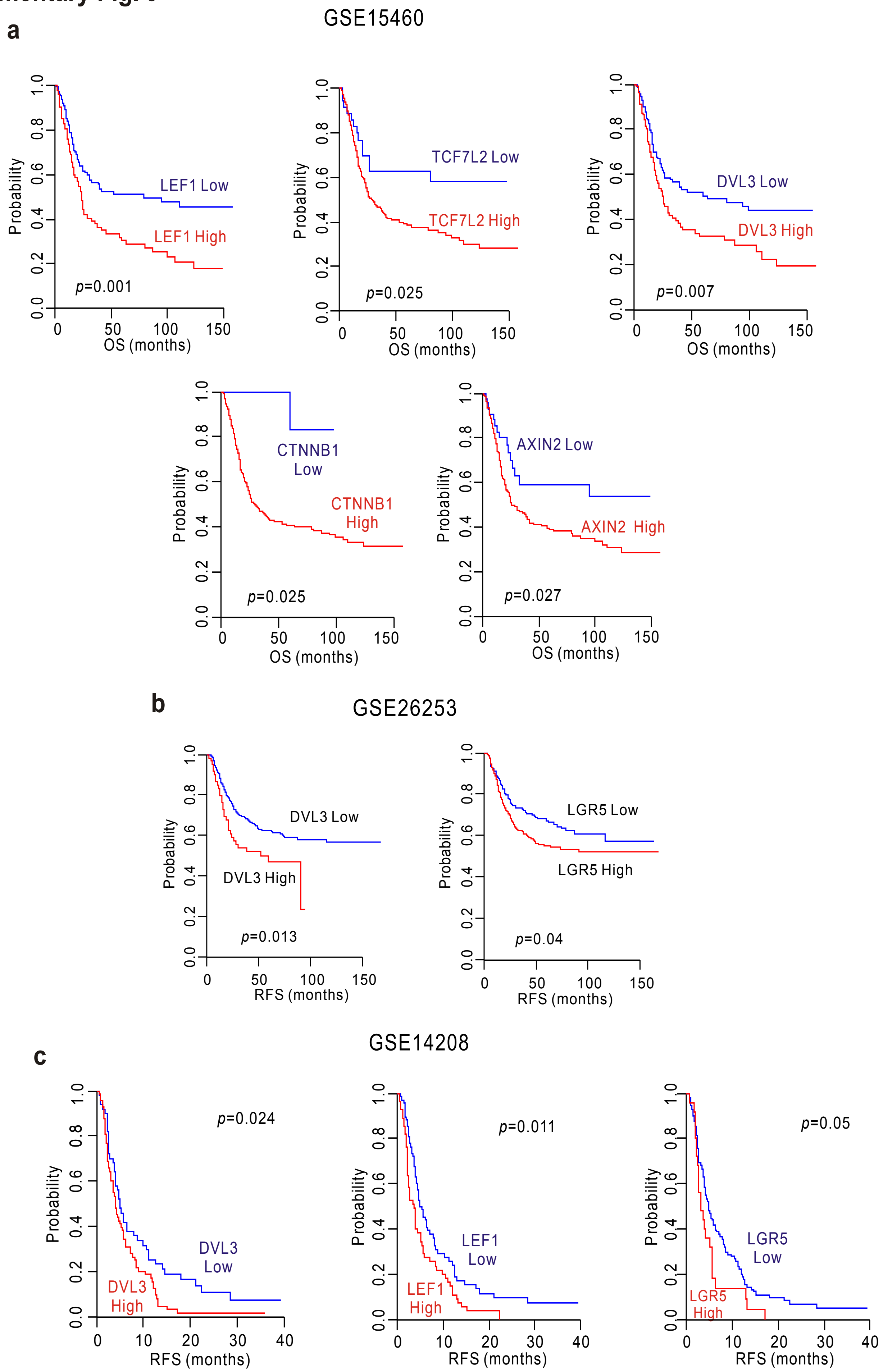

Supplementary Fig. 9 | Kaplan-Meier plots of Wnt-target genes in gastric cancer

a-c, Kaplan-Meier plots of indicated gastric cancer patients from indicated gene expression profile (GSE15460, GSE26253, GSE14208). When patients were classified according to the expression level of indicated Wnt-target genes, the OS or RFS of patients with higher expression of indicated genes were significantly worse than those of patients with lower expression of indicated genes. Kaplan-Meier plots and the log-rank test were used to estimate patient prognosis.

Supplementary Fig. 10

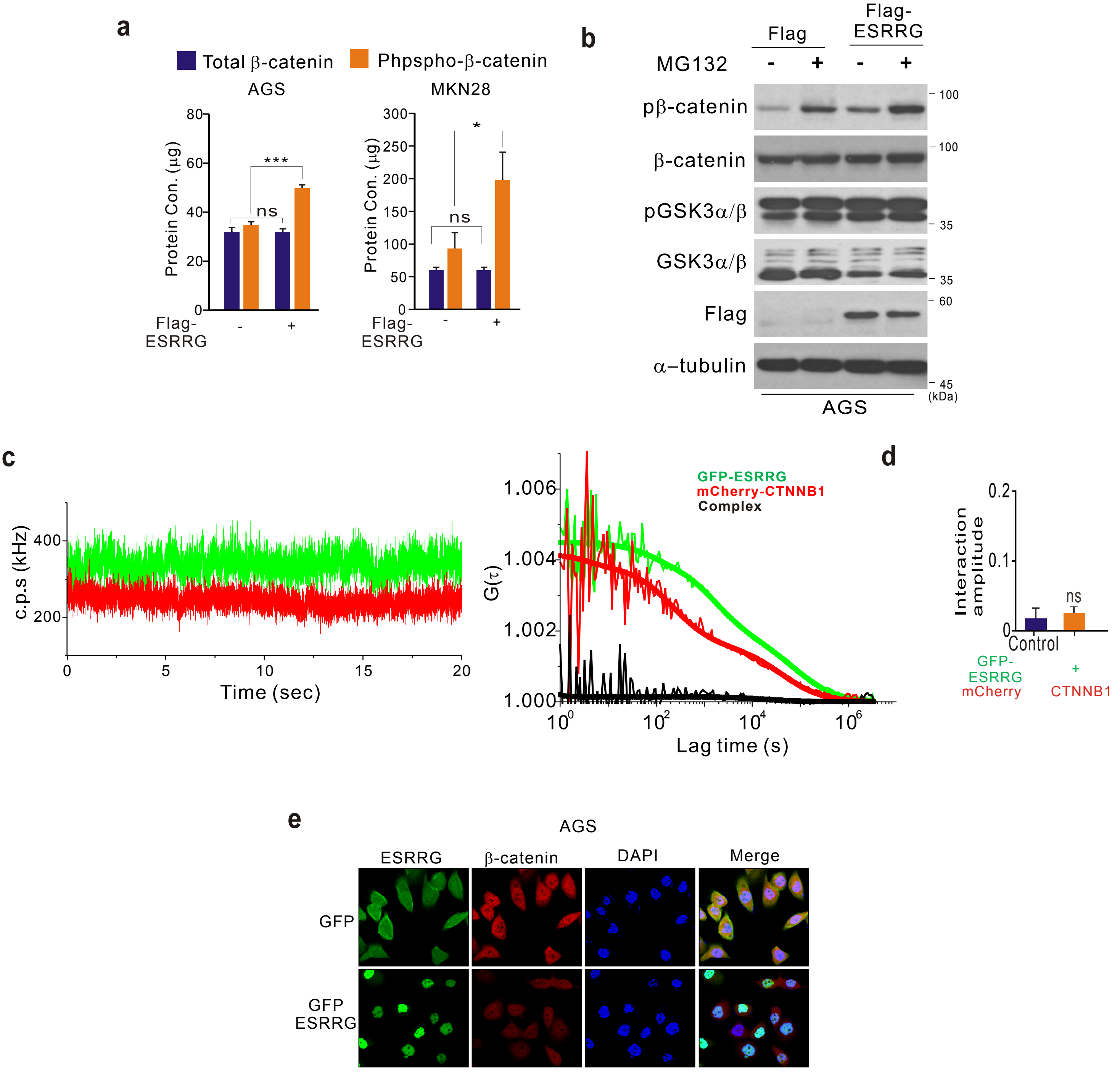

Supplementary Fig. 10 | ESRRG function on Wnt-signaling in gastric cancer cell

a and b, Indicated gastric cancer cells were stably infected with ESRRG lenti-viral vector (Flag-ESRRG) or control lenti-vial vector (Flag) and the cells were used for ELISA analyzed or western blot. c, changes over time of the average fluorescence intensities (count per second; c.p.s in kHz) of GFP-ESRRG and mCherry-CTNNB1, and the corresponding correlation functions, are shown. Changes over time of the average fluorescence intensity and the corresponding correlation functions obtained in cells co-expressing monomer GFP and mCherry are also shown. d, summary of protein interaction amplitudes. The interaction amplitude represents the mean value of the relative cross-correlation amplitude. e, infected cells were used for cellular imaging using confocal microscope. Student t-test (two-tailed) was applied to estimate the significance of gene expression changes: *, $p < 0.05$; **, $p < 0.01$; ***, $p < 0.005$

Supplementary Fig. 11

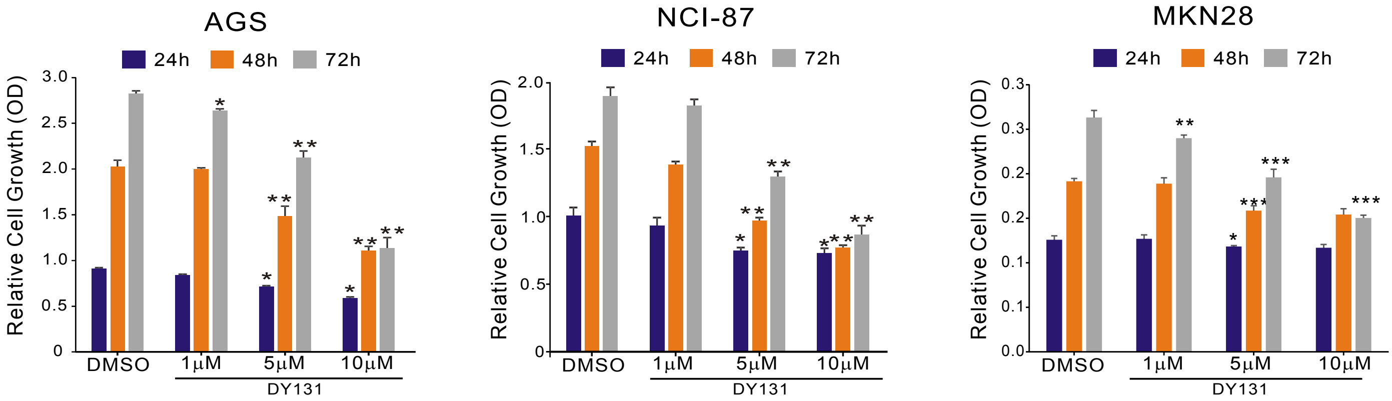

Supplementary Fig. 11 | Cell proliferation by ESRRG agonist DY131 in gastric cancer cells

DY131 was treated to indicated gastric can cells and CCK8 was done at the indicated time. Student t-test (two-tailed) was applied to estimate the significance of gene expression changes: *, $p < 0.005$, **, $p<0.001$

Supplementary Fig. 12

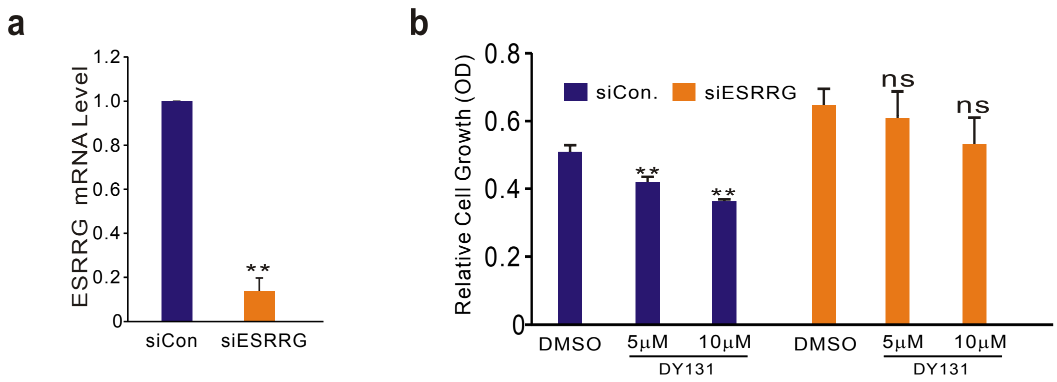

Supplementary Fig. 12 | Dy131 is ESRRG specific agoinist.

a, siESRRG (or siCon) was treated to SNU638 cells and cell lysates was used for qRT-PCR to examine the ESRRG expression. b,after siESRRG (or siCon.) was treated to the cells, DY131 was treated to the cells for 48 hrs and CCK8 was done. Student t-test (two-tailed) was applied to estimate the significance of gene expression changes: *, *p* < 0.005, **, *p*<0.001

Supplementary Fig. 13

a

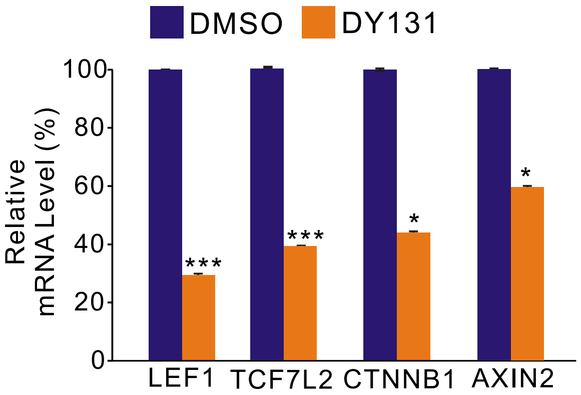

b

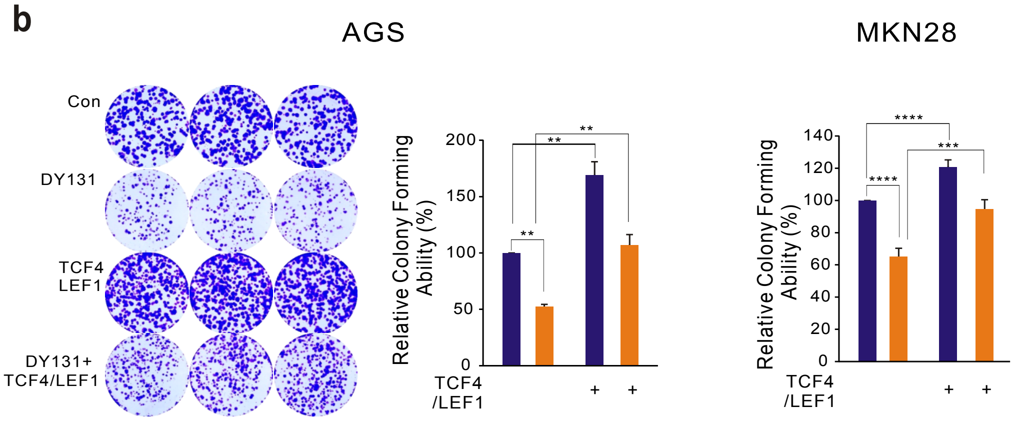

Supplementary Fig. 13 | Gene expression alteration by DY131 in gastric cancer cells

a, DY131 (5μM) was treated to MKN28 gastric cancer cells for 72 hrs and the cell lysates was used for qRT-PCR using gene-specific primers as indicated.
b,DY131 was treated to AGS and MKN28 cell and indicated plasmid was transfected with GC cells for 48 hrs. After 10 days, colony forming assay was done and quantified with Image J. Student t-test (two-tailed) was applied to estimate the significance of gene expression changes: *, $p < 0.05$ **, $p < 0.01$, ***, $p < 0.005$

Supplementary Fig. 14

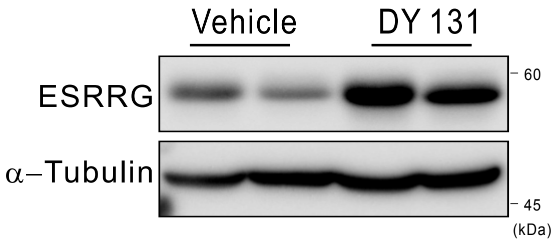

Supplementary Fig. 14 | ESRRG expression by DY131 in xenograft model.

DY131 was injected to gastric cancer xenograft model and tissues were collected for western blot analysis with indicated antibody.

Supplementary Fig. 15

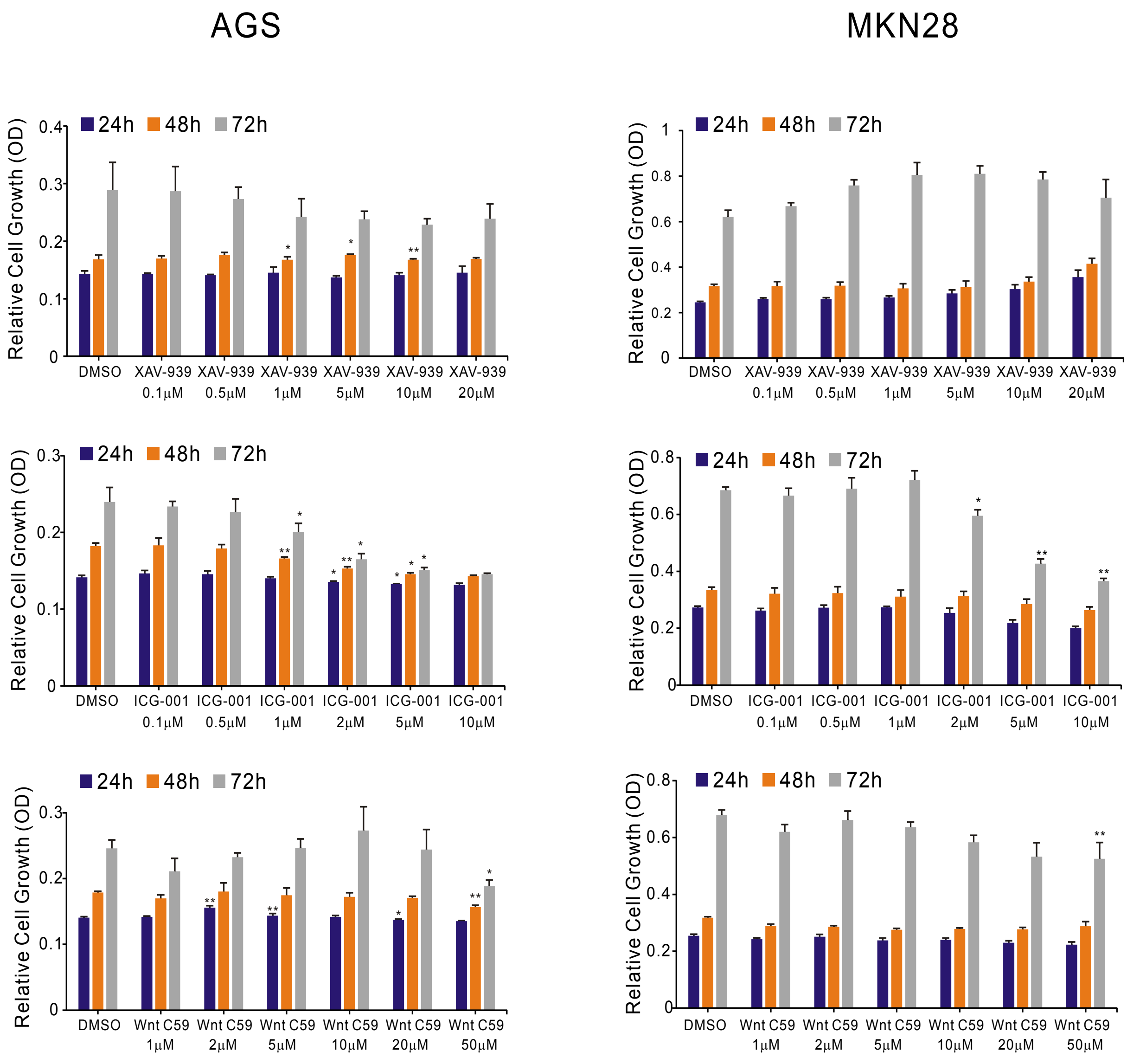

Supplementary Fig. 15 | Cell proliferation by WNT antagonist in gastric cancer cells

Indicated WNT-antagonist was treated to indicated gastric can cells and CCK8 was done at the indicated time. Student t-test (two-tailed) was applied to estimate the significance of gene expression changes: *, $p < 0.005$, **, $p < 0.001$

Supplementary Fig. 16a. The original full scan of immunoblot utilized in Figure 1d

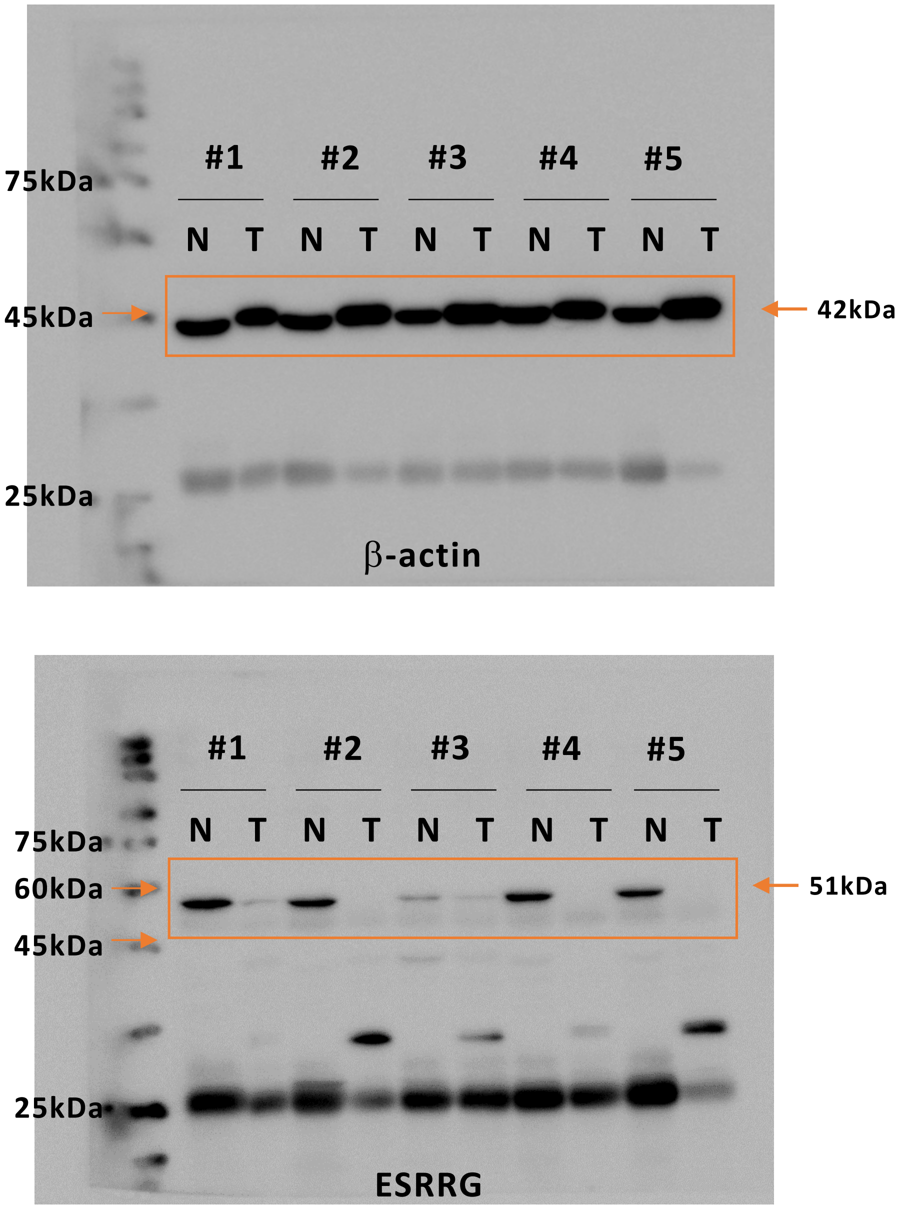

Supplementary Fig. 16b. The original full scan of immunoblot utilized in Figure 2e

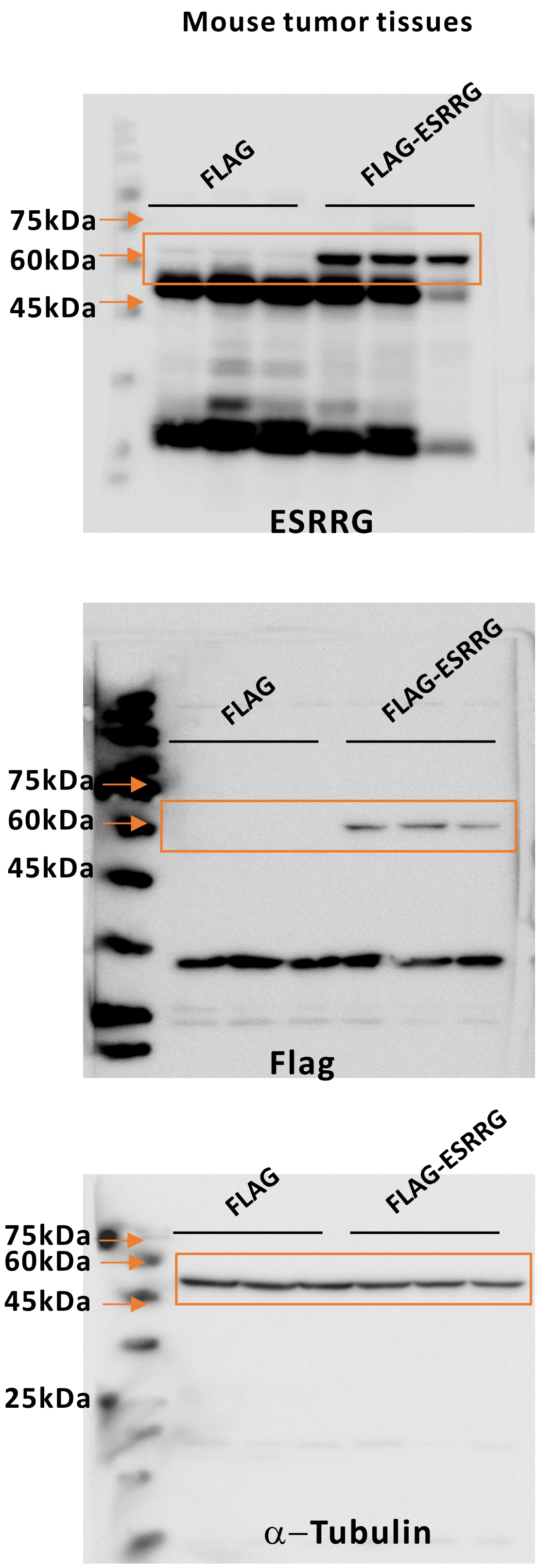

Supplementary Fig. 16c. The original full scan of immunoblot utilized in Figure 5a

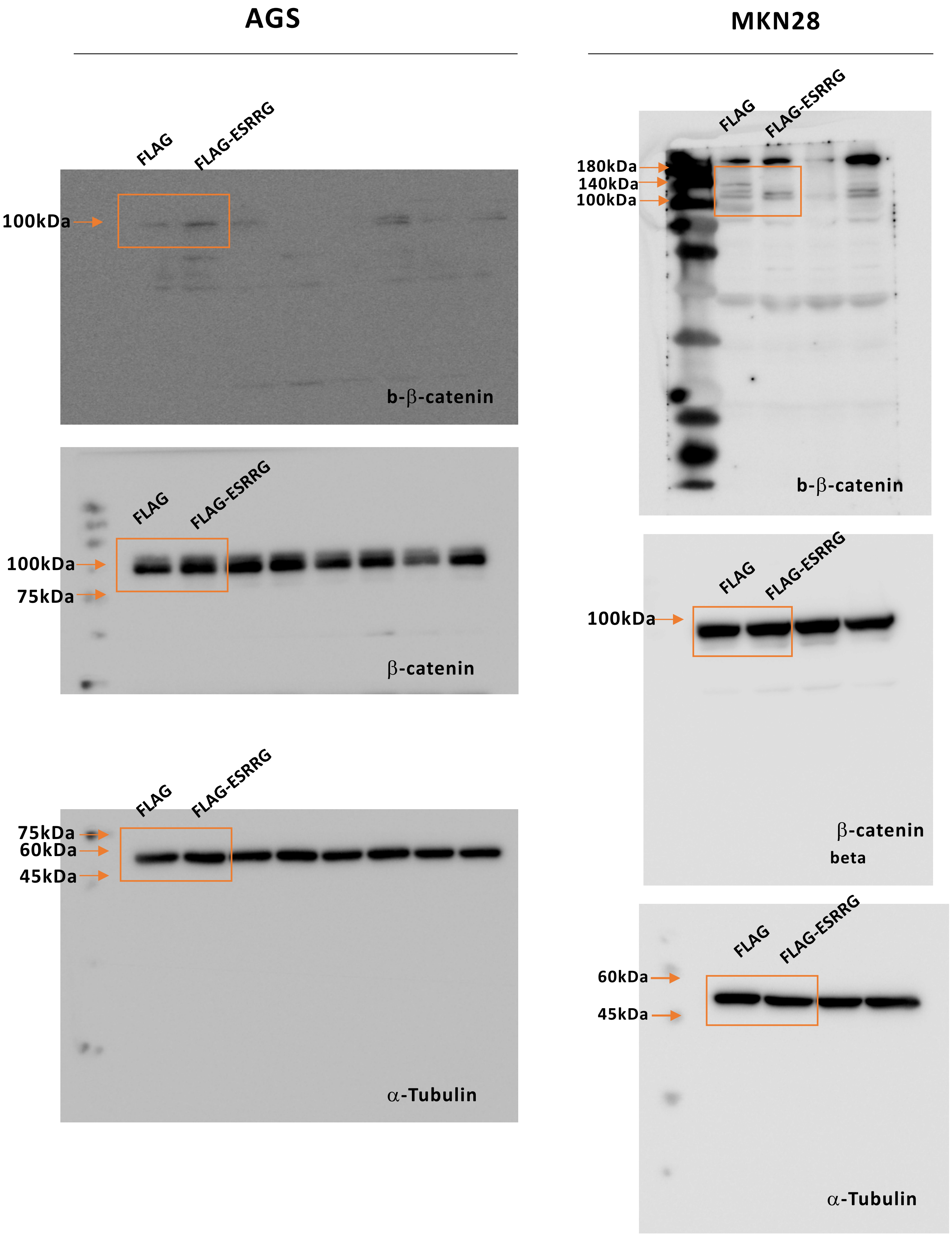

Supplementary Fig. 16d. The original full scan of immunoblot utilized in Figure 5b

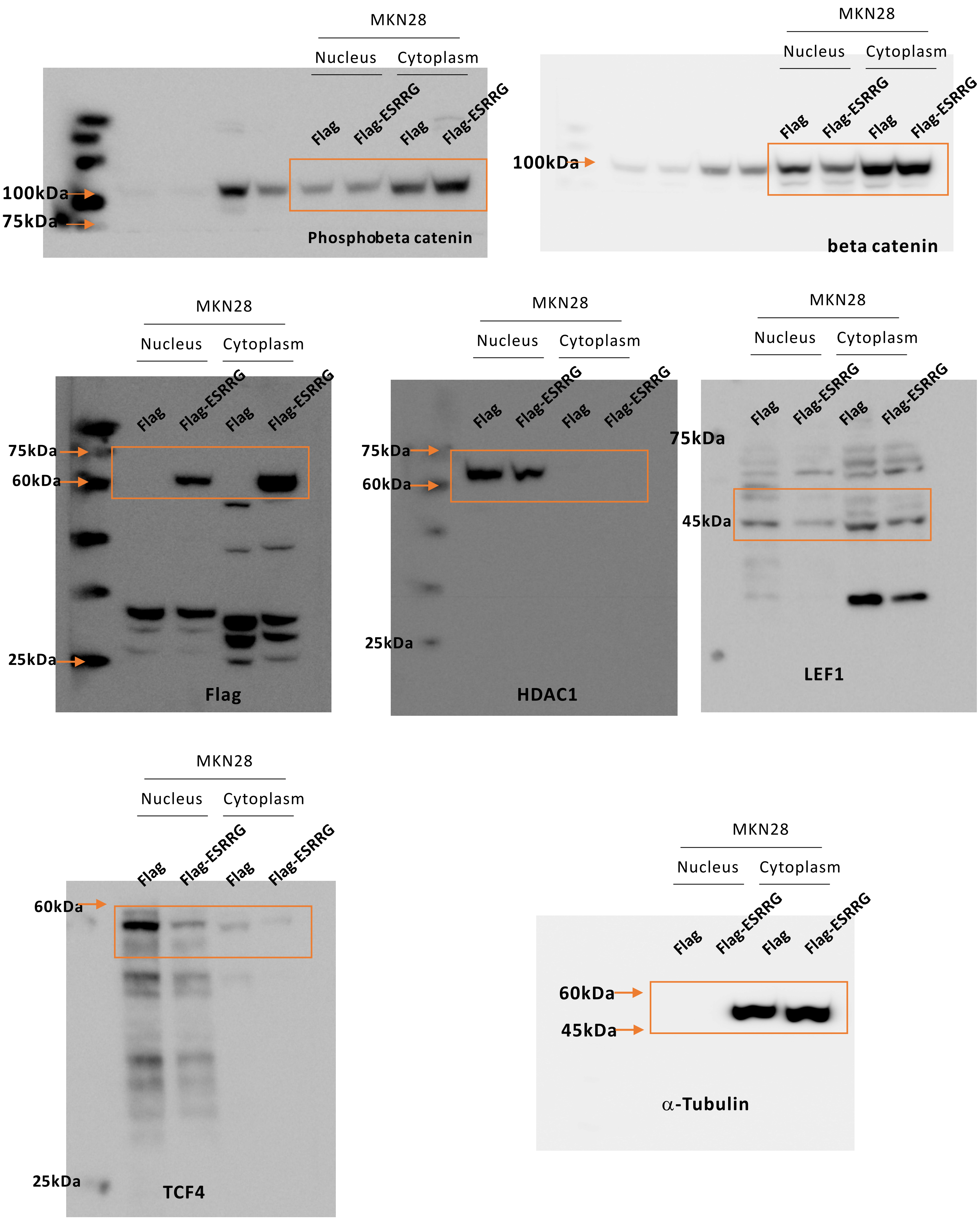

Supplementary Fig. 16e. The original full scan of immunoblot utilized in Figure 5c

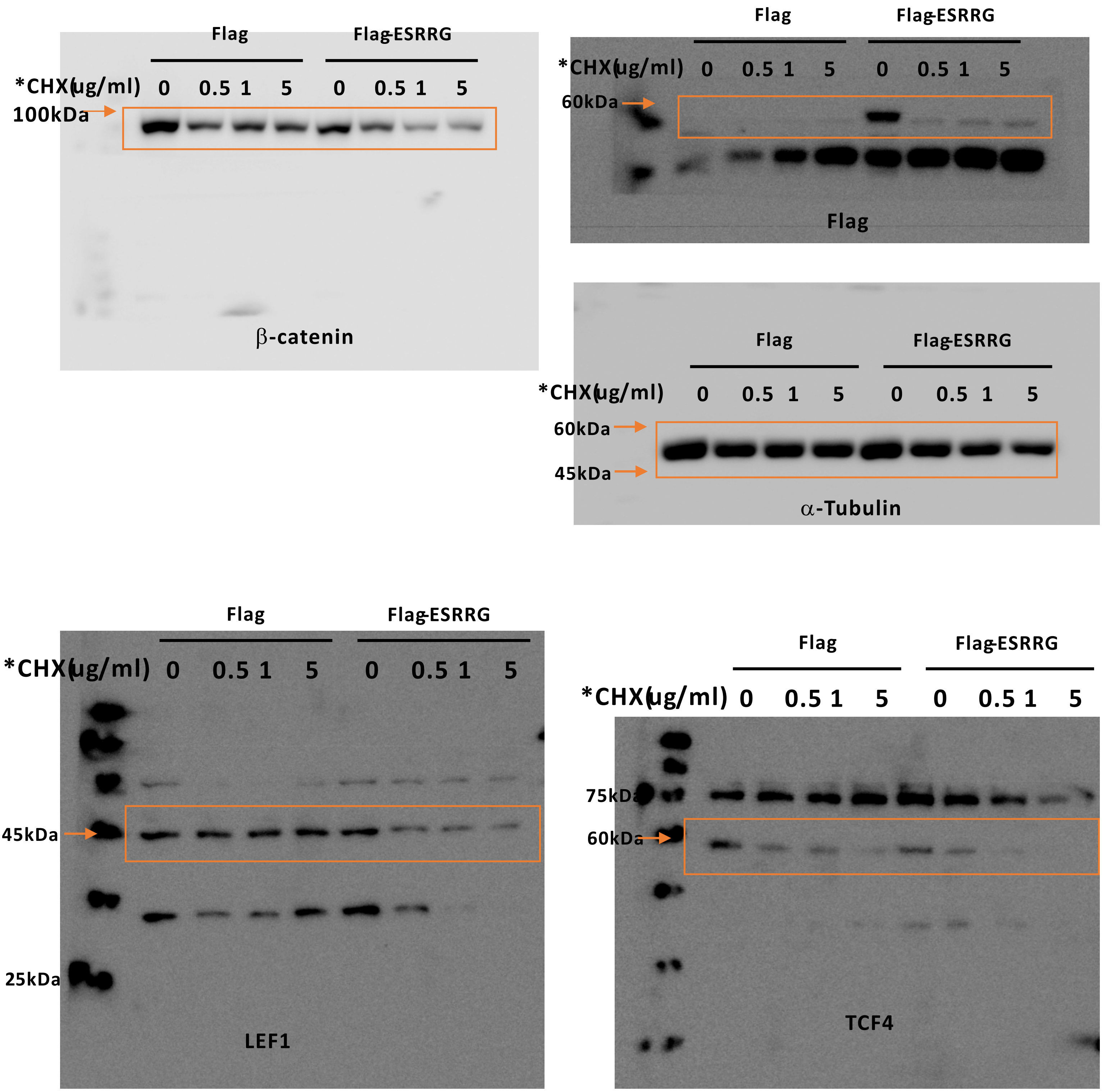

Supplementary Fig. 16f. The original full scan of immunoblot utilized in Figure 5d

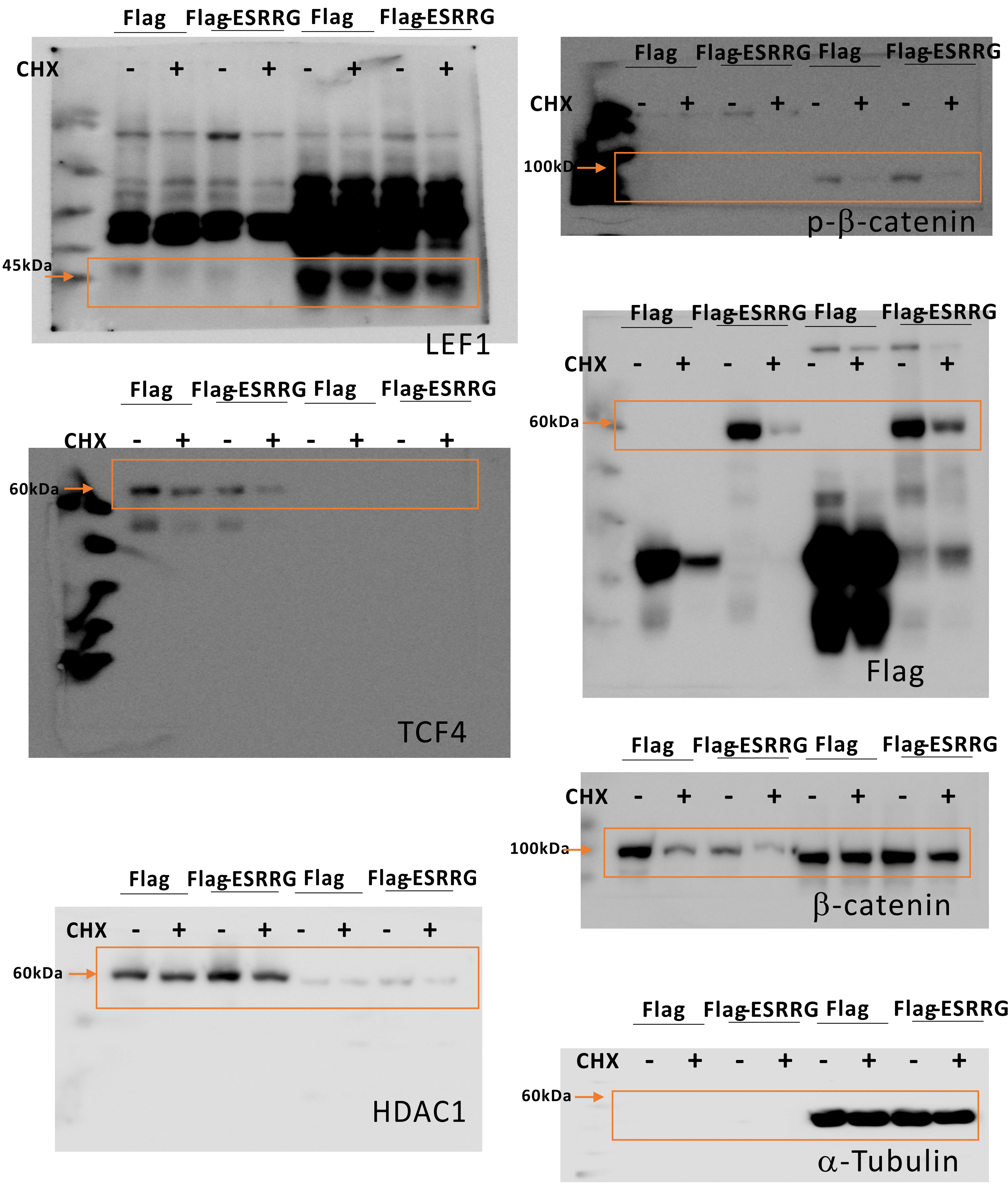

Supplementary Fig. 16g. The original full scan of immunoblot utilized in Figure 5g

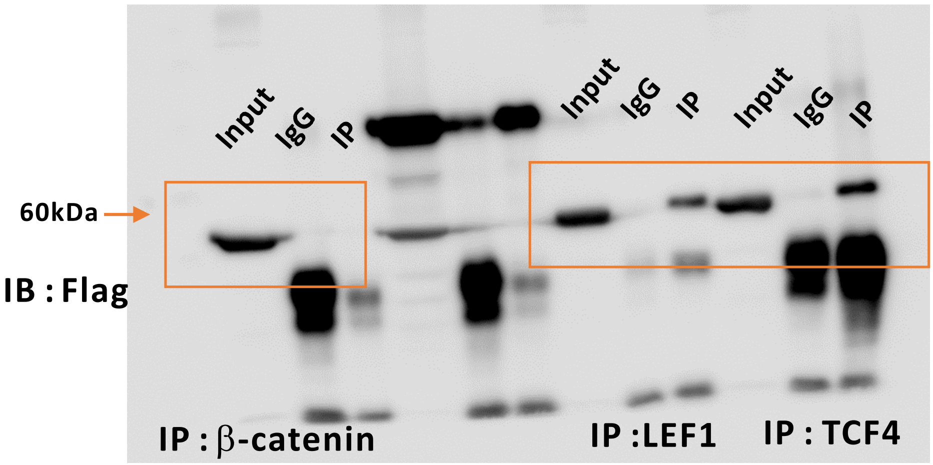

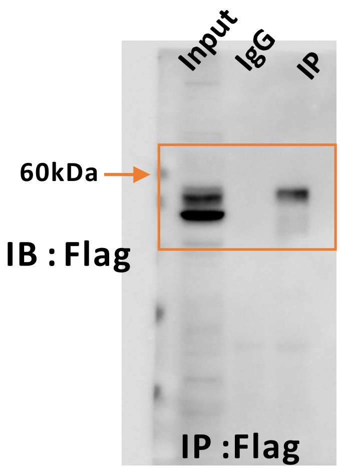

Supplementary Fig. 16h. The original full scan of immunoblot utilized in Supplementary Figure 3a

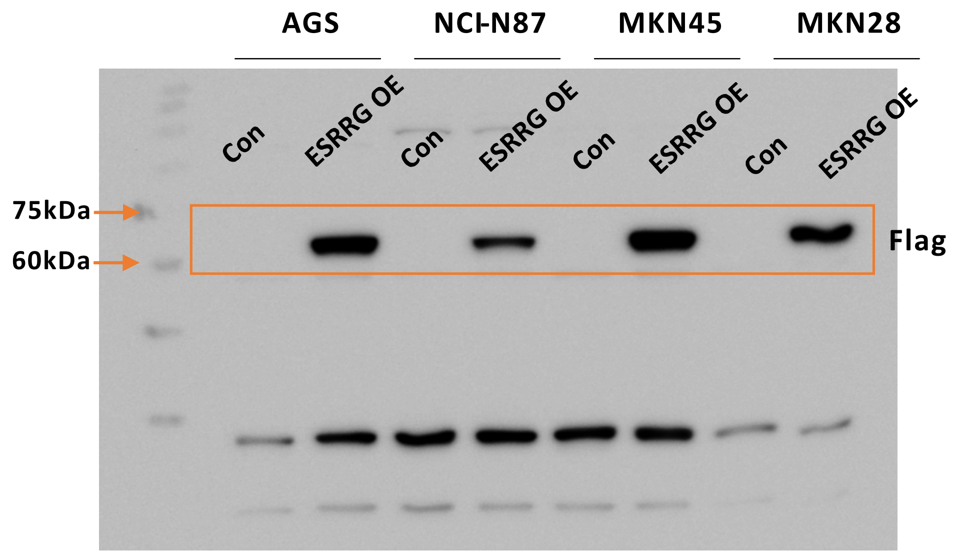

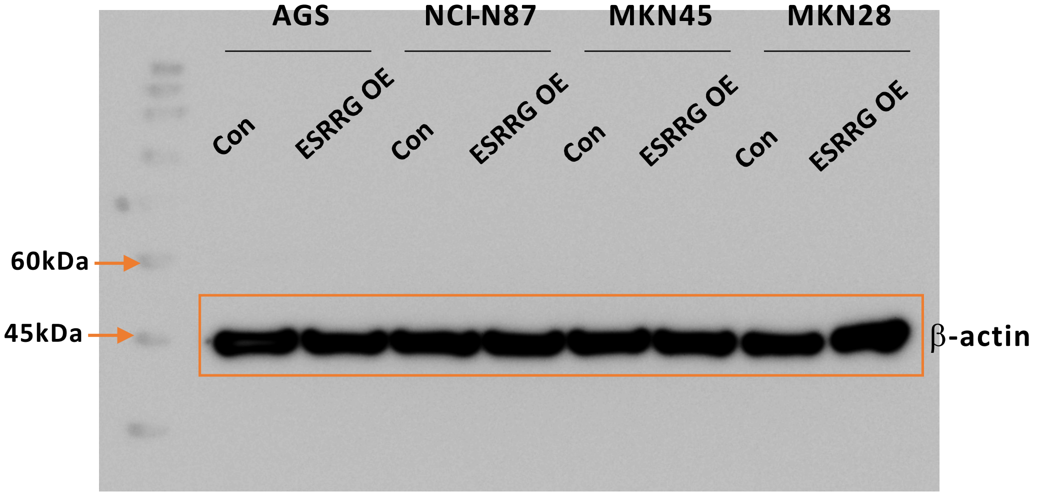

Supplementary Fig. 16i. The original full scan of immunoblot utilized in Supplementary Figure 10

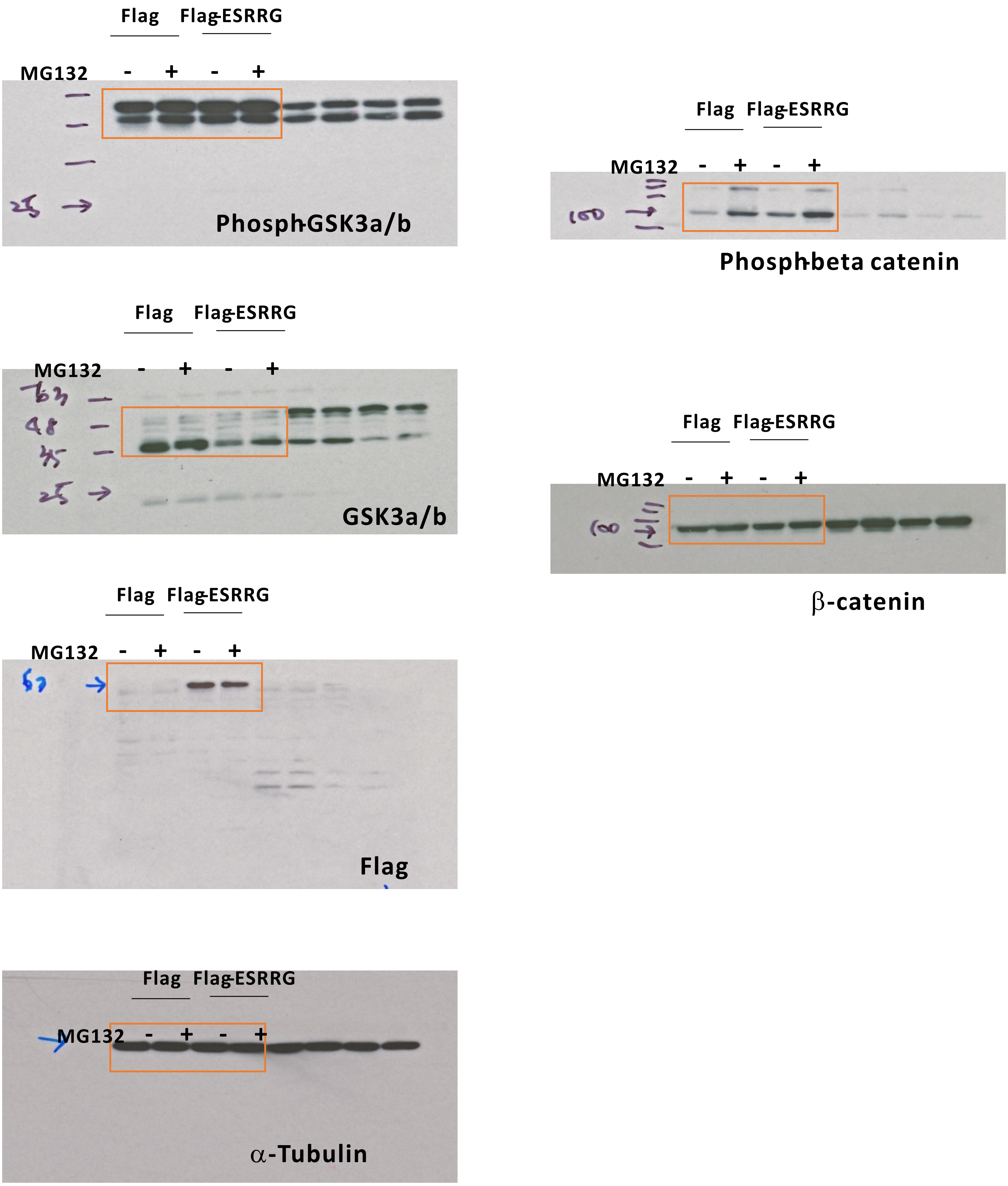

Supplementary Fig. 16j. The original full scan of immunoblot utilized in Supplementary Figure 14

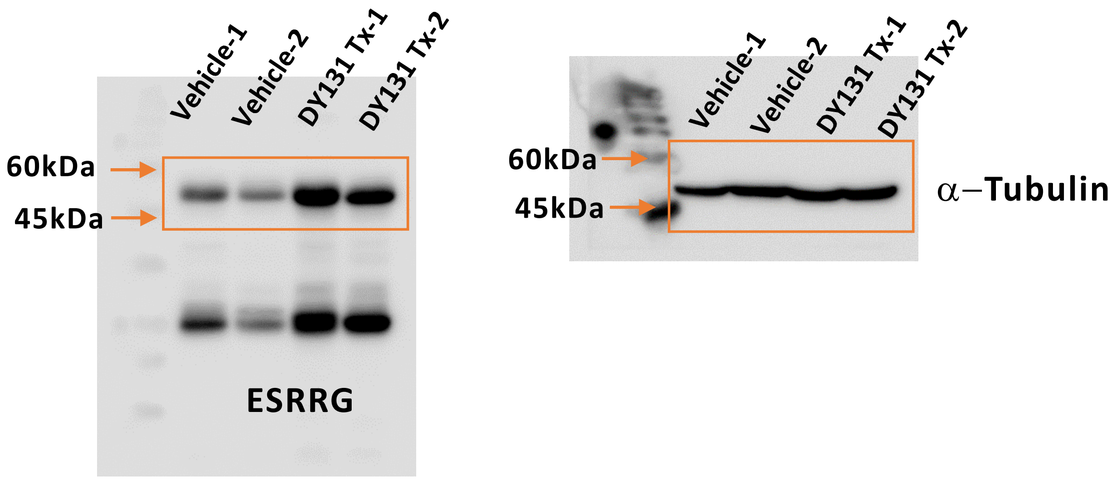

Supplement: Supplementary file 1 — Supplementary Information [file 41467_2018_4244_MOESM1_ESM.pdf]
